# Dupuytren’s and Ledderhose Diseases in a Family with *LMNA*-Related Cardiomyopathy and a Novel Variant in the *ASTE1* Gene

**DOI:** 10.3390/cells6040040

**Published:** 2017-11-01

**Authors:** Michael V. Zaragoza, Cecilia H. H. Nguyen, Halida P. Widyastuti, Linda A. McCarthy, Anna Grosberg

**Affiliations:** 1 UCI Cardiogenomics Program, Department of Pediatrics, Division of Genetics & Genomics, School of Medicine, University of California, Irvine, CA 92697, USA; cecilihn@uci.edu (C.H.H.N.); hwidyast@uci.edu (H.P.W.); 2 Department of Biological Sciences, School of Medicine, University of California, Irvine, CA 92697, USA; 3 Department of Biomedical Engineering and The Edwards Lifesciences Center for Advanced Cardiovascular Technology, University of California, Irvine, CA 92697, USA; mccarthl@uci.edu (L.A.M.); grosberg@uci.edu (A.G.)

**Keywords:** *LMNA* gene, lamin, cardiomyopathy, arrhythmias, Dupuytren’s disease, Ledderhose disease, fibromatosis, genetic susceptibility, *ASTE1* gene

## Abstract

Dupuytren’s disease (palmar fibromatosis) involves nodules in fascia of the hand that leads to flexion contractures. Ledderhose disease (plantar fibromatosis) is similar with nodules of the foot. While clinical aspects are well-described, genetic mechanisms are unknown. We report a family with cardiac disease due to a heterozygous *LMNA* mutation (c.736C>T, p.Gln246Stop) with palmar/plantar fibromatosis and investigate the hypothesis that a second rare DNA variant increases the risk for fibrotic disease in *LMNA* mutation carriers. The proband and six family members were evaluated for the cardiac and hand/feet phenotypes and tested for the *LMNA* mutation. Fibroblast RNA studies revealed monoallelic expression of the normal *LMNA* allele and reduced lamin A/C mRNAs consistent with *LMNA* haploinsufficiency. A novel, heterozygous missense variant (c.230T>C, p.Val77Ala) in the Asteroid Homolog 1 (*ASTE1*) gene was identified as a potential risk factor in fibrotic disease using exome sequencing and family studies of five family members: four *LMNA* mutation carriers with fibromatosis and one individual without the *LMNA* mutation and no fibromatosis. With a possible role in epidermal growth factor receptor signaling, *ASTE1* may contribute to the increased risk for palmar/plantar fibromatosis in patients with Lamin A/C haploinsufficiency.

## 1. Introduction

Dupuytren’s disease (DD) or palmar fibromatosis is characterized by abnormal proliferation of fibroblasts in the palmar fascia and leads to progressive and disabling contractures of the fingers [1]. Initially, a palmar nodule is observed which is followed by cord formation as the disease progresses. Disease pathogenesis is complex and involves activation of fibroblasts to myofibroblasts, production of several profibrotic cytokines including transforming growth factor beta 1 (TGF-beta1), transforming growth factor alpha (TGF-alpha) and epidermal growth factor (EGF), dysregulation of signaling pathways including mitogen-activated protein kinase (MAPK), Akt, and Wnt/beta-catenin signaling pathways, and increased extracellular matrix proteins including collagen I and III [2]. Treatment includes surgical excision but there is a high risk of relapse [3]. Ledderhose disease (LD) or plantar fibromatosis is a rarer disease that affects the plantar fascia as nodules and cords formed along the tendons of the foot, which can lead to painful walking or standing with disease progression [4,5,6].

Family studies have shown that there is a genetic component with manifestation of these diseases. About 40% of those affected with DD have an affected relative and a family history of the disease is correlated with increased number of affected fingers and increased frequency of extrapalmar involvement [7]. Although inheritance is heterogeneous, DD is most often transmitted in an autosomal dominant fashion with variable penetrance; a similar hereditary pattern may be possible for LD since it is considered the plantar counterpart of Dupuytren’s disease [8,9,10]. Given the low prevalence of the disease [5], the genetics of LD has not yet been fully characterized and the genetic mechanism of both diseases remains largely unknown.

The *LMNA* gene encodes for Lamin A/C, intermediate filament proteins that serve as structural components crucial in maintaining cell nucleus integrity [11,12]. Due to its adjacency to the nuclear inner membrane and proximity to the genome, Lamin A/C has also been implicated in key nuclear functions including cell differentiation, regulation of gene expression through chromatin localization and remodeling, and activation or repression of signaling pathways [12,13,14,15,16,17]. Over 400 *LMNA* mutations have been reported in patients with phenotypes known collectively as laminopathies including premature aging syndromes (Hutchinson–Gilford Progeria Syndrome and atypical Werner syndrome), skeletal muscle myopathies (Emery-Dreifuss Muscular Dystrophy and Limb-Girdle Muscular Dystrophy), and cardiac diseases (dilated cardiomyopathy and cardiac conduction defects) [11,18]. While musculoskeletal features have been frequently noted in *LMNA*-associated diseases, DD has been reported only once in a patient with *LMNA*-associated atypical Werner syndrome [19]. Comorbidity of DD and Limb-Girdle Muscular Dystrophy has been reported in another patient; however, the three most common *LMNA* mutations were not detected in the patient [20].

Here, we report a unique family with cardiac disease due to a previously reported nonsense mutation in the Lamin A/C gene (*LMNA* c.736C>T (p. Gln246Stop)) and variable features of DD and LD. After fibroblast RNA studies revealed a mechanism consistent with *LMNA* haploinsufficiency, we used whole exome sequencing, bioinformatics analysis, and family studies to identify a novel, heterozygous missense variant in the Asteroid Homolog 1 gene (*ASTE1* c.230T>C, p.Val77Ala) as a candidate susceptibility variant in fibromatosis for patients with *LMNA* haploinsufficiency and a potential genetic factor in the etiology of DD and LD.

## 2. Materials and Methods

### 2.1. Study Family

#### 2.1.1. Pedigree and Consent

**Figure 1:**
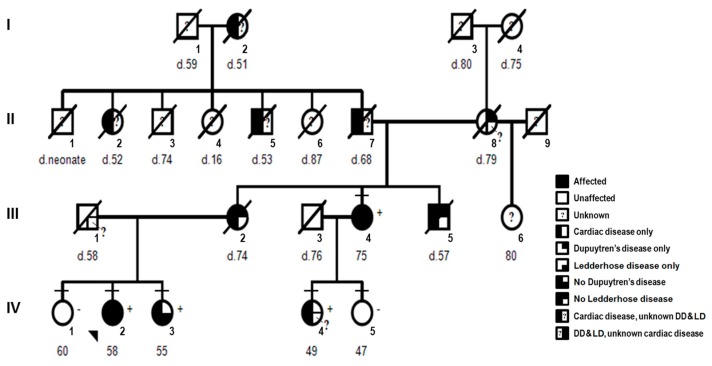
Pedigree: *LMNA*-related cardiac disease, Dupuytren’s disease (DD), and Ledderhose disease (LD). Squares denote males and circles females with current age below. Short horizontal lines mark individuals consented in the study. Diagonal lines indicate deceased individuals with age of death (d). The proband (IV-2) is designated with an arrow. Complete-filled symbols indicate cardiac disease (cardiomyopathy, arrhythmia or sudden death), Dupuytren’s disease, and Ledderhose disease. Left half-filled indicate only cardiac disease and right half-filled symbols indicate only Dupuytren’s disease (upper right) or only Ledderhose disease (lower right). Individuals with unknown or unconfirmed clinical status are noted with a question mark. The presence (+) or absence (−) of the *LMNA* c.736C>T (p.Gln246Stop) is provided. One additional family member (Patient 3) was removed after consent to publish was declined.

A family (Figure 1) with English, Scottish, Irish, German and Native American ancestry was studied. All subjects gave their informed consent for inclusion before they participated in the study. The study was conducted in accordance with the Declaration of Helsinki, and the protocol was approved by the University of California institutional review board (#2011-8030). Pedigree, completed questionnaire on health history, medical records, and samples (saliva and/or skin biopsies) were obtained for each individual. Additional signed consent was obtained prior to manuscript submission. One family member (Patient 3) was removed from the pedigree after consent to publish was declined.

#### 2.1.2. Clinical Features

Each individual was classified by clinical status as affected, unaffected or unknown for heart disease, DD and LD (Figure 1). Classification for heart disease was as described previously [21]. For DD, individuals were classified as affected if they displayed physical signs of fibromatosis in their hands including dimpling, tethering or puckering of palmar skin, flexion contracture with limited extension of the fingers, subcutaneous palmar nodules or cords [3]. For LD, individuals were classified as affected with foot involvement including plantar nodules, cords or limited toe movement [5,6]. Risk factors associated with DD were also evaluated including smoking, alcohol abuse and diabetes [3]. For deceased individuals, family history and review of available medical records were used to determine the status of cardiac disease, DD, and LD.

#### 2.1.3. Proband

The proband (IV-2, Figure 1) is a 58-year-old female who was healthy until age 45 when she had syncope and an irregular heartbeat. Holter studies showed periods of sinus arrest. She was diagnosed with Sick Sinus Syndrome and a dual chamber pacemaker was placed. She had a normal echocardiogram (Ejection Fraction (EF) = 50% (normal > 45%), Interventricular Septal (IVS) thickness = 11 mm (normal 7–12 mm), Left Ventricular Posterior Wall (PW) thickness, LVPW = 8 mm (normal 7–12 mm), Left Ventricular End Diastolic Diameter (LVEDD) = 53 mm (normal 35–55 mm), Fractional Shortening (FS) = 25% (normal > 25%)). At age 55, she developed worsening fatigue and dyspnea on exertion and was found to have atrial fibrillation, nonsustained ventricular tachycardia, and high-degree atrioventricular block. Echocardiogram showed reduced systolic function (EF = 33%, LVEDD = 52 mm). Due to these findings plus her family history, clinical genetic testing for dilated cardiomyopathy (DCM) was performed and the results showed a heterozygous nonsense mutation in *LMNA* exon 4 (c.736C>T, p.Gln246Stop). The pacemaker was replaced with biventricular implantable cardioverter defibrillator (ICD). At her current age, she was doing well with no shortness of breath, palpitations or syncope and no ICD shocks. Her medications include carvedilol, losartan, and warfarin, and her most recent echocardiogram showed mild–moderately impaired LV systolic function with normal LV size and wall thickness (EF = 40–45%, IVS = 10 mm, LVPW = 7 mm, LVEDD = 50 mm, FS = 28%).

**Figure 2:**
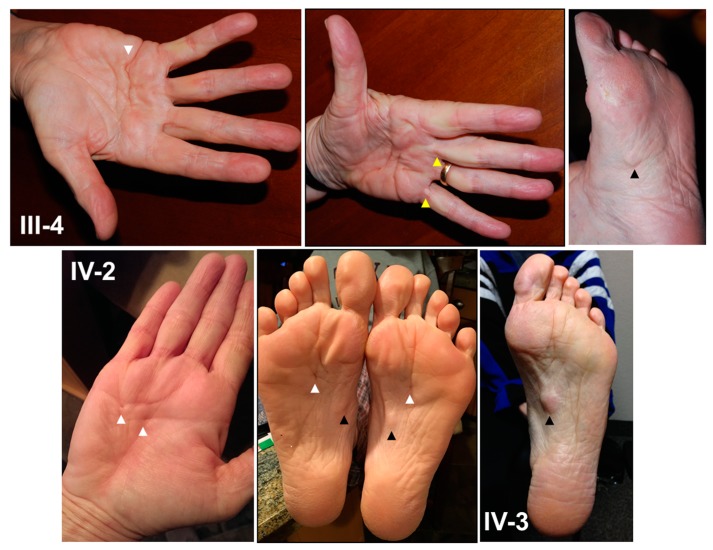
Dupuytren’s and Ledderhose disease. Photographs: (top panel) right hand, left hand, and left foot of the aunt (**III-4**), (bottom panel, left-middle) right hand and feet of the proband (**IV-2**) and (bottom panel, right) left foot of the sister (**IV-3**) with skin tethering/puckering or dimpling (white arrows), cord formation (yellow arrows), and central medial plantar nodules (black arrows). An image from one additional family member (Patient 3) was removed after consent to publish was declined.

At age 50, she noticed early signs of DD. She developed right hand stiffness after driving her car for extended periods and noted skin dimpling on her right palm (Figure 2). At age 55, she developed two plantar nodules (less than 1 cm in diameter), one each at the central medial region of her right and left soles consistent with LD (Figure 2). At her present age of 58, she denied hand or foot pain. She has no cord formation, contractures or knuckle pads of her hands. She has full extension of her fingers and toes with no limitations in function. She noted limited alcohol use (1 glass of wine/week) and no tobacco use. She had a normal birth and development with no signs or symptoms of neuromuscular disease.

#### 2.1.4. Affected Family Members

There are at least 12 family members affected with cardiac disease, DD, and/or LD (Figure 1): five living (one maternal aunt (III-4), one sister (IV-3), and one maternal first cousin (IV-4) and seven deceased individuals (I-2, II-2, II-5, II-7, II-8, III-2, III-5). Clinical features for one affected living family member with cardiac disease and DD (Patient 3) were removed after additional consent to publish was declined. The maternal grandfather (II-7) had an irregular heart beat diagnosed in his 40s, pacemaker implantation at age 64, heart failure at age 66, and cardiac arrest at age 68. With sudden death of two siblings (II-2, II-5) and his mother (I-2) all in their 50s, *LMNA*-related cardiac disease was likely introduced into the family by the maternal grandfather (II-7). DD was likely introduced into the family by the maternal grandmother (II-8), the only individual with known DD without cardiac disease. The main clinical features are provided in Table S1, Supplementary Material.

### 2.2. Molecular Studies

#### 2.2.1. Sanger Sequencing for *LMNA* Exon 4

DNA sequencing and analysis of *LMNA* exon 4 were done first using saliva DNA as described [21]. The presence (+) or absence (−) of the *LMNA* nonsense mutation (c.736C>T, p.Gln246Stop) was tested for seven individuals: five affected with cardiac disease (III-4, proband: IV-2, IV-3, IV-4, and Patient 3) and two unaffected (IV-1 and IV-5) family members.

#### 2.2.2. *LMNA* RNA and DNA Studies in Fibroblast

To evaluate the molecular mechanism of the *LMNA* mutation, primary cultures were established using skin biopsies obtained from six individuals, four affected (patients: P1 (IV-4), P2 (IV-3), P3, and P4 (III-4)) and two unaffected (controls: C1 (IV-5) and C2 (IV-1)) family members. Fibroblasts were not available for the proband (IV-2). In addition, skin fibroblast from a healthy individual (CC-251, Lonza Group Ltd., Basel, Switzerland) was used as an unrelated control. Total RNA and genomic DNA extractions, qualitative RNA analysis using targeted PCR amplifications of cDNA, Sanger sequencing, and PCR primers used to evaluate for exonic variants in *LMNA* were conducted as described [21]. Real-time quantitative PCR (RT-qPCR) was also performed on cDNA from P1 (IV-4) and P2 (IV-3) and their gender-age matched controls, C1 (IV-5) and C2 (IV-1), respectively. P3 and P4 (III-4) were not included in the RT-qPCR studies due to absence of matched controls from the family. Pre-designed KiCqStart™ Primers (Sigma-Aldrich, Saint Louis, MO, USA) for Lamin A, Lamin C, and Beta macroglobulin (*B2M*) transcripts and the ViiA7 Real-Time PCR system (Applied Biosystems, Carlsbad, CA, USA) were used to amplify the cDNA samples in triplicate. Ct values were analyzed using the comparative Ct method to obtain relative gene expression (RGE) for Lamin A and C. Student’s *t*-test was used to determine statistical significance, with *p* < 0.05 considered as statistically significant.

#### 2.2.3. Exome Sequencing, Bioinformatics Analysis, and Variant Filtering

For four family members, genomic DNA isolated from skin fibroblast was sent to DNA Link USA, Inc. (San Diego, CA, USA) for whole exome sequencing (WES). These studies included three affected individuals with cardiac disease, DD and/or LD (III-4, IV-3 and Patient 3) and one unaffected individual without cardiac disease, DD and LD (IV-1). The proband (IV-2) was not included in the exome sequencing studies since fibroblast were not available. The WES studies used enrichment by Agilent SureSelect XT Human All Exon V5 library and paired-end sequencing by Illumina HiSeq2500 at 100X coverage. The CLC bio Biomedical Genomics Workbench v3.5 (Qiagen, Valencia, CA, USA) was used to align the data to the human genome reference (hg38). The Identify and Annotate Variants (WES-HD) workflow and variant analysis tools for splicing effects were utilized to identify DNA variants. The lists of variants for each of the four family members were filtered in a step-wise approach to identify potential mutations and modifiers similar to the approach as described [21,22]. Candidate variants were identified as potentially deleterious single nucleotide variants (SNV) and insertion–deletion variants (indels) that were shared among the three affected family members (III-4, IV-3 and Patient 3) and not shared with the one individual unaffected at age 60 years old (IV-1). Using the ExAC human genome database [23], the candidates were ranked by allele frequency to identify rare variants (allele frequency < 1%) as high-priority candidates.

#### 2.2.4. Family Studies

To validate the exome sequencing results and to further evaluate the high-priority candidate variants as potential genetic factors in DD and LD, family studies of five individuals using saliva or fibroblast DNA were conducted by Sanger sequencing to determine co-segregation of each candidate variant and phenotype. These studies included four affected individuals with cardiac disease, DD and/or LD (III-4, proband: IV-2, IV-3 and Patient 3) and one unaffected individual without cardiac disease, DD and LD (IV-1). Due to questionable phenotype and age-related penetrance for DD and LD, exome sequencing and family studies were not done for two family members, a 49-year-old female (IV-4) heterozygous for the *LMNA* mutation with no hand signs of DD and questionable foot findings for LD and a 47-year-old female (IV-5) without the *LMNA* mutation and no signs of DD or LD. Primer sequences and PCR conditions are available by request.

## 3. Results

### 3.1. LMNA DNA and RNA Studies

**Figure 3:**
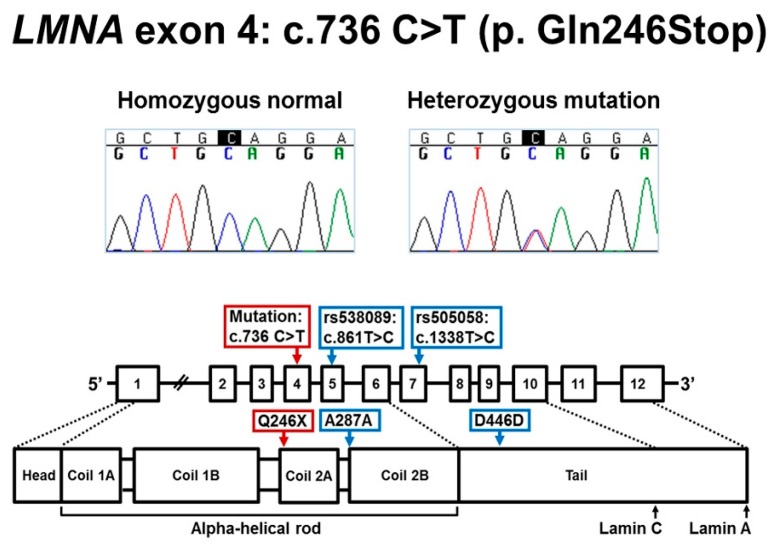
*LMNA* c.736C>T (p.Gln246Stop). **Top panel** shows the Sanger sequencing chromatograms: normal allele for unaffected individuals and heterozygous mutation for affected individuals. **Bottom panel** depicts the location of the nonsense mutation and two expressed polymorphisms rs538089 (c.861T>C, Ala287Ala) and rs505058 (c.1338T>C, Asp446Asp) in the mRNA and Lamin A/C protein.

First, DNA sequencing of *LMNA* exon 4 was conducted for seven family members (Figure 1 and Figure 3). All five individuals (III-4, proband: IV-2, IV-3, IV-4 and Patient 3) affected with cardiac disease were heterozygous for *LMNA* nonsense mutation (NM_170707.3: c.736C>T, p.Gln246Stop). The mutation is predicted to result in a stop codon at amino acid 246 and premature truncation of the Lamin A/C protein. The mutation was absent in both unaffected individuals (IV-1 and IV-5).

**Figure 4:**
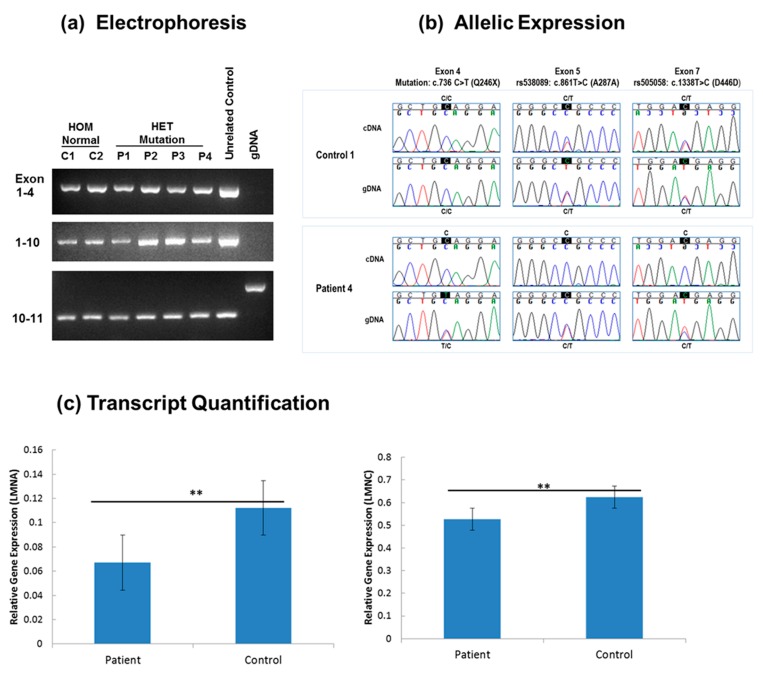
Expression analysis of *LMNA* in patient and control fibroblasts. (**a**) Electrophoresis: cDNA fragment analysis of *LMNA* mRNA (Exon 1–4, Exon 1–10, and Exon 10–11) for fibroblasts from two unaffected family members: C1 (IV-5) and C2 (IV-1) (HOM Normal: lanes 1–2), four patients: P1 (IV-4), P2 (IV-3), P3 and P4 (III-4) (HET Mutation: lanes 3–6), and an unrelated control (lane 7). Genomic DNA served as a negative control (lane 8). Only the expected products were observed with cDNA amplification (lanes 1–7): 898 bp for Exon 1–4, 1886 bp for Exon 1–10, and 297 bp for Exon 10–11. No products were observed with gDNA amplification (lane 8) for Exon 1–4 and Exon 1–10. For Exon 10–11, the expected 1041 bp product was observed with gDNA (lane 8) and was absent with cDNA amplification (lanes 1–7); (**b**) Allelic Expression: Sanger sequencing chromatograms from genomic DNA and cDNA for control C1 (IV-5) and patient P4 (III-4). For control fibroblasts, biallelic expression (C/T) in the cDNA and heterozygosity for C/T in the genomic DNA is shown at variants rs538089 in exon 5 and rs505058 in exon 7. In contrast, detection of only the normal C allele at the mutation site in exon 4 and single C alleles at rs538089 and rs505058 demonstrates monoallelic expression for patient fibroblasts; (**c**) Lamin A/C transcript quantification: RT-qPCR results from patient and control cDNA measuring the relative transcript level of Lamin A and Lamin C. Results shown are the mean transcript levels ± error (SEM) of patient: P1 (IV-4), P2 (IV-3) and control: C1 (IV-5) and C2 (IV-1). For both Lamin A and C, there is a significant reduction of transcribed mRNA in patient fibroblasts compared to control fibroblasts (*n* = 6, ** *p* < 0.05).

Next, to evaluate the molecular mechanism of the *LMNA* mutation, RNA and DNA studies were done on mutant (patient) and normal (control) skin fibroblasts derived from four affected and two unaffected family members, respectively. The qualitative and sequence analyses comparing *LMNA* mRNA in fibroblast showed only the expected sequences and no differences in cDNA fragment length consistent with normal mRNA splicing (Figure 4a). To determine which *LMNA* allele was expressed, two heterozygous exonic variants, rs538089 (NM_170707.3: c.861T>C, Ala287Ala) located in exon 5 and rs505058 (NM_170707.3: c.1338T>C, Asp446Asp) in exon 7, detected in our fibroblast DNA sequencing for all 12 exons were examined in cDNA. Comparison of *LMNA* sequences from cDNA and genomic DNA showed that both C and T alleles for each variant (rs538089 and rs505058) were detected in all control fibroblasts (Figure 4b). In contrast, only the C alleles for each variant (rs538089 and rs505058) and the normal C allele at the mutation site in exon 4 were detected in all patient fibroblasts (Figure 4b). These findings were consistent with the normal C allele in exon 4 being in cis with both C alleles in exons 5 and 7 and supported monoallelic expression of the normal *LMNA* allele (Figure 4b). In addition, these results confirmed the presence of only the single heterozygous *LMNA* mutation for patient fibroblasts and the absence of *LMNA* mutations in the control fibroblasts. To confirm Lamin A/C haploinsufficiency in patients, RT-qPCR was performed on patients and controls fibroblasts. Relative gene expression (RGE) quantification showed a reduction of Lamin A and Lamin C transcripts in patient fibroblasts compared to control fibroblasts (Figure 4c). For Lamin A, RGE was 0.067 ± 0.012 in patient fibroblast and 0.112 ± 0.009 in control fibroblast, a 1.6-fold reduction of gene expression. For Lamin C, RGE was 0.526 ± 0.033 in patient fibroblast and 0.624 ± 0.038 in control fibroblast, a 1.2-fold reduction.

### 3.2. Exome Sequencing and Co-Segregation for DD/LD-Associated Variants

**Figure 5:**
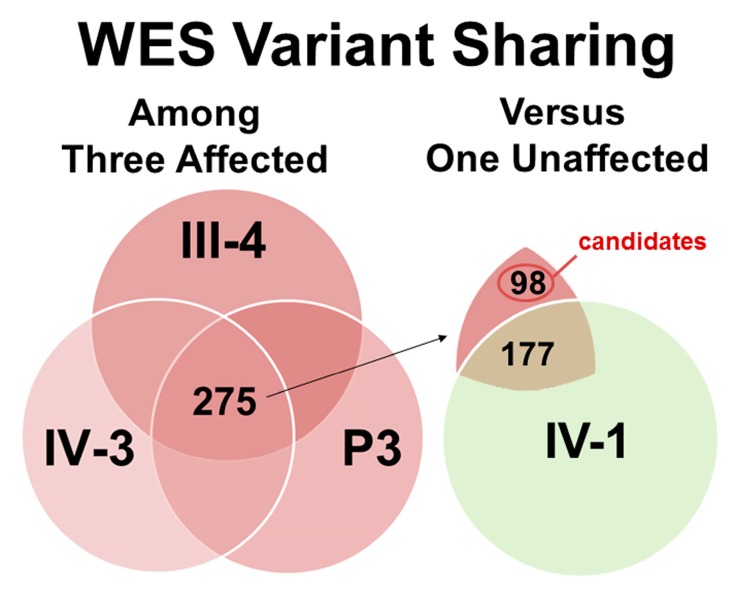
Whole Exome Sequencing (WES) Studies and Variant Sharing of Four Family Members to Identify Candidate Variants in DD and LD. Diagram provided to represent the total number of potentially deleterious variants found in the three affected individuals: 1677 variants for III-4, 1681 variants for IV-3, and 1815 variants for Patient 3 (red circles) and the one unaffected individual: 1,761 variants for IV-1 (green circle). For the affected individuals, the central overlapping region represents the shared variants (*n* = 275). When compared to the variants of the unaffected individual, 177 shared variants (overlapping region) were excluded and 98 unshared variants (non-overlapping region) were identified as candidates in DD and LD.

Results of the WES studies from four family members: III-4, IV-3 and Patient 3 (affected) and IV-1 (unaffected) are provided in Figure 5 and Table S2, Supplementary Material. On average per sample, exome sequencing resulted in 8.1 gigabases of data (90.4% > Quality Score (Q30)) from 107.2 million reads. Bioinformatics analysis showed 99.4% of the reads mapped to hg38 for each sample and on average per sample, 81X mean depth of coverage of the target bases with 85.8% having mean coverage of at least 30X. Variant calling analysis identified on average, 20,416 total high-quality, unique variants with 12,874 heterozygous variants per sample. Of these heterozygous variants, an average of 6488 variants were predicted to result in non-synonymous amino acids changes with 1734 of these variants located at highly conserved sites (phastCons score > 0.90). Exome data is available from the National Center for Biotechnology Information (NCBI) Sequence Read Archive under NCBI BioProject “Human Exome Sequencing in LMNA Cardiomyopathy” (Accession: PRJNA320422, ID: 320422).

To identify candidate variants in DD and LD, the four lists of heterozygous, non-synonymous, highly conserved variants were compared between the three affected family members (III-4, IV-3 and Patient 3) and one unaffected family member (IV-1) (Figure 5). Among the three affected family members, 275 variants were shared. Of these, 98 variants were identified as candidates and 177 variants were excluded when compared to the list of variants found in the one unaffected family member (Figure 5).

To identify high-priority candidates for further evaluation, the 98 candidates were ranked by allele frequency (Table S3, Supplementary Material). Seven novel or rare variants were identified including the expected *LMNA* mutation c.736C>T (p.Gln246Stop), three novel SNVs: *ASTE1* c.230T>C (p.Val77Ala), *FZD2* c.535A>C (p.Thr179Pro) and *FSD1* c.608G>A (p.Arg203Gln), two known SNVs: *OR51A7* c.763A>G (p.Ile255Val) and *SYPL2* c.272A>T (p.Tyr91Phe), and one known indel *USF3* c.4416_4418delGCA (p.Gln1478del). The *USF3* variant was not further evaluated because it is an in-frame deletion of one codon and the clinical significance of the variant is likely benign (ClinVar variant #218482).

The remaining five high-priority candidates were further evaluated by Sanger sequencing in family studies using one additional affected family member (proband: IV-2) (Table 1). Only two of the five high-priority candidates were validated, shared by all four affected individuals (III-4, proband: IV-2, IV-3 and Patient 3), and unshared with the one unaffected individual (IV-1). These two variants included a novel missense variant (NM_001288950: c.230T>C, p.Val77Ala) in the Asteroid Homolog 1 gene (*ASTE1*), and a rare known missense variant (NM_001004749: c.763A>G, p.Ile255Val) in the Olfactory Receptor Family 51 Subfamily A Member 7 gene (*OR51A7*). These results also excluded three of the five high-priority candidates: *FSD1* c.608G>A (p.Arg203Gln), *FZD2* c.535A>C (p.Thr179Pro) and *SYPL2* c.272A>T (p.Tyr91Phe). The *FSD1* variant was excluded because it was not found in the proband (IV-2) (Table 1). The *FZD2* and *SYPL2* variants were excluded because of the lack of validation by Sanger sequencing. Review of the WES data found the discrepancies consistent with a false-negative result for *SYPL2* variant in the unaffected individual (IV-1) and false-positive results for *FZD2* variant in the affected individuals (III-4, IV-3 and P3).

## 4. Discussion

### 4.1. Molecular Mechanism of LMNA c.736C>T (p.Gln246Stop)

We report *LMNA* c.736C>T (p.Gln246Stop), a previously described nonsense mutation as the primary mutation in a family with typical *LMNA*-related cardiac disease that includes DCM, cardiac conduction defects, and sudden death [24,25,26]. To investigate the molecular mechanism by which *LMNA* c.736C>T (p.Gln246Stop) may lead to disease, we conducted RNA studies on patient and control fibroblasts. These results were consistent with haploinsufficiency due to monoallelic expression of the normal *LMNA* allele in mutant fibroblasts and elimination of aberrant mRNAs transcribed from the mutated allele by nonsense-mediated mRNA decay (NMD). These results in fibroblasts concur with a previous study using gene and protein expression in blood and myocardial samples [27].

Lamin A/C haploinsufficiency may lead to cardiac disease in our family as the result of nuclear dysfunction such as abnormal cell differentiation, altered gene expression, and dysregulation of cell signaling [13,14,15,16,17]. Defects in Lamin A/C have been shown specifically to dysregulate the TGF beta 1, MAPK, mTOR, and Wnt/ beta-catenin signaling pathways [15,16,17,28,29]. *LMNA* mutation in a mouse model was associated with increased MAPK activity in response to stress that resulted in aberrant expression of target genes and with decreased Wnt/beta-catenin activity that supports the involvement of both signaling in the pathogenesis for lamin-cardiomyopathy [15,17]. The role of Lamin A/C haploinsufficiency in cell differentiation has been suggested in studies of mouse embryonic stem cells that showed that the levels of Lamin A/C were important in lineage-specific differentiation [30].

### 4.2. Dupuytren’s and Ledderhose Diseases as Primary Features of Laminopathies?

We also report the novel occurrence of DD and LD in *LMNA* c.736C>T (p.Gln246Stop) DCM patients. Our studies found that four (III-4, IV-2, IV-3 and Patient 3) of five family members heterozygous for the mutation had DD and/or LD along with typical *LMNA*-related cardiac disease. Severe DD that required surgery was noted for two deceased family members (III-2 and III-5) who were obligate carriers for the mutation. The co-occurrence of DD and a *LMNA* mutation has been previously reported in a patient with atypical Werner syndrome, a 31-year-old male with mild DD and a heterozygous missense mutation, *LMNA* c.898 G>A (p.Asp300Asn) [19]. These findings suggest that both palmar and plantar fibromatosis could be a part of the primary laminopathy phenotype, extra-cardiac features with clinical variability.

However, we argue that the presence of DD and LD in our family may be instead due to additional genetic factors and not primarily due to the *LMNA* mutation. First, our pedigree analysis indicated that while the *LMNA*-related cardiac disease was introduced into the family by the maternal grandfather (II-7), DD was introduced by the proband’s maternal grandmother (II-8), the only DD-affected individual with no cardiac disease. Furthermore, patients previously reported to carry the same nonsense mutation as our family, or other premature truncation mutations with mechanisms consistent with Lamin A/C haploinsufficiency, did not have DD or LD [21,24,25,27]. *LMNA* c.736C>T (p.Gln246Stop) has been previously described in two families with DCM and atrioventricular block that includes an Italian proband [24] and a Polish 40-year-old male proband with a family history of heart failure [25]; however, extra-cardiac features were not reported. These observations suggest that DD is not exclusive to laminopathy patients and additional genetics factors in family members heterozygous for the *LMNA* mutation may increase the susceptibility to develop DD along with the primary laminopathy phenotype of cardiac disease.

### 4.3. Identification of a Candidate Susceptibility Variant for DD and LD in the ASTE1 Gene

To identify potential genetics factors in the etiology DD and LD, we performed WES on three family members heterozygous for the *LMNA* mutation and affected with cardiac disease, DD and/or LD (III-4, IV-3 and Patient 3) and one unaffected family member without the *LMNA* mutation and unaffected with cardiac disease, DD and LD (IV-1). Bioinformatics analysis and variant filtering was used to identify 98 total candidate variants and five high-priority variants based on allele frequencies (Table S3). Family studies by Sanger sequencing were performed to validate the WES results and to further evaluate the high-priority candidate using a fourth affected family member with cardiac disease, DD and LD (proband: IV-2) (Table 1). In addition to the *LMNA* mutation c.736C>T (p.Gln246Stop), only two variants co-segregated with the fibromatosis phenotype: a novel missense variant in the Asteroid Homolog 1 gene (*ASTE1* NM_001288950: c.230T>C, p.Val77Ala) and a rare known missense variant in the Olfactory Receptor Family 51 Subfamily A Member 7 gene *OR51A7* NM_001004749: c.763A>G, p.Ile255Val).

Our results provide initial evidence for *ASTE1* c.230T>C (p.Val77Ala) as a candidate susceptibility variant for DD and LD and a potential role for the *ASTE1* gene in the etiology of fibromatosis. The *ASTE1* gene is located at chromosome 3q22.1 and encodes for the protein asteroid homolog 1 (Uniprot entry: ASTE1_HUMAN, accession No. Q2TB18) [31,32]. Although there is limited information available, the *ASTE1* gene is expressed in skin tissue and the ASTE1 protein has 679 amino acids with a possible role in epidermal growth factor receptor (EGFR) signaling by sequence similarity to the asteroid gene in Drosophila [31,33]. The *ASTE1* c.230T>C (p.Val77Ala) variant is located in the Xeroderma Pigmentosum complementation group G (XPG) N-terminal domain (AA 1-96) and PIN domain-like (AA 1-207) of the ASTE1 protein with inferred functions of nuclease activity and in DNA repair [34]. No primary disease-associated mutations have been reported for *ASTE1* [31,35]; however, secondary mutations in *ASTE1* were detected in colorectal cancers with microsatellite instability [36]. Thus, functional effects of *ASTE1* c.230T>C (p.Val77Ala) relevant to DD and LD are possible and warrant further investigation. No support was found for the *OR51A7* variant or human olfactory receptor gene family after review of the literature and databases [31,37].

With the possible role of *ASTE1* in EGFR signaling [33], the complex pathway with significant roles in cell–cell communication, cell fate, and proliferation [38], our findings raise the possibility that *ASTE1* c.230T>C (p.Val77Ala) may result in susceptibility to palmar and plantar fibromatosis in our family. EGFR signaling has been implicated in the pathogenesis of fibromatosis in previous studies of diseased tissues from patients with DD [2,39,40,41]. Co-expression of EGFR protein and TGF-alpha has been found in proliferating myofibroblasts in palmar fibromatosis nodules of DD patients [39]. A synergistic effect of EGF and TGF-beta1 on fibroblast proliferation in patients with DD compared to normal fibroblast was found [40]. In addition, as an indicator of receptor activation, a higher ratio of surface-to-intracellular EGFR in palmar fascia was found during the progression of disease in DD patients [41].

## 5. Conclusions

In summary, we report the novel co-occurrence of palmar and plantar fibromatosis and *LMNA*-related cardiac disease in a family with a previously described nonsense mutation, *LMNA* c.736C>T (p.Gln246Stop). *LMNA* expression studies in fibroblasts support haploinsufficiency as the primary molecular mechanism due to monoallelic expression of the normal *LMNA* allele. To identify additional genetic factors that may contribute to the pathogenesis of fibromatosis in the setting of Lamin A/C haploinsufficiency, we used exome sequencing and family studies to identify *ASTE1* c.230T>C (p.Val77Ala) as a candidate susceptibility variant for DD and LD and a possible role for the *ASTE1* gene in the etiology of fibromatosis. Since Lamin A/C haploinsufficiency may disrupt biological processes also important in the etiology of fibromatosis including cell differentiation, gene expression, and signaling pathways, additional defects in these processes due to *ASTE1* c.230T>C (p.Val77Ala) may lead to susceptibility for fibrotic disease in *LMNA* heterozygotes.

We may only speculate about the relevance of *ASTE1* c.230T>C (p.Val77Ala) at this time. Since all four family members affected with fibromatosis were double heterozygotes for the *LMNA* mutation c.736C>T (p.Gln246Stop) and *ASTE1* c.230T>C (p.Val77Ala), it is possible that both Lamin A/C haploinsufficiency and an *ASTE1* defect are required for disease. Alternatively, *ASTE1* c.230T>C (p.Val77Ala) may act as an independent susceptibility variant. Thus, future research studies include functional analysis of our patient fibroblast cells for altered cell signaling (e.g., EGFR and MAPK pathways) associated with increased cell proliferation, profibrotic gene expression, and ECM production. Ultimately, disease modeling utilizing induced pluripotent stem cells (iPSC) derived from our patient fibroblast cells may be used to investigate the effects of *LMNA* and *ASTE1* defects in lineage-specific cell proliferation and differentiation to mesodermal derivatives such as myofibroblast. Elucidation of the precise molecular pathogenesis of fibrosis in the *LMNA* mutation background may confer knowledge on the role of Lamin A/C in maintaining physiological balance for a functional organism as the basis to develop treatments for disease.

## Figures and Tables

**Table 1:** Family studies of the high-priority variants to identify genetic factors in DD and LD ^a^.

Gene ^b^	Position (hg38)	DNA Variant	Protein Change	dbSNP ^c^	ExAC Frequency ^d^	Family Studies: Genotype ^e^				
						III-4	IV-2	IV-3	P3	IV-1
*ASTE1*	Chr3:131025077	c.230T>C	p.Val77Ala	novel	No data	−/+	−/+	−/+	−/+	−/−
*FZD2*	Chr17:44558223	c.535A>C	p.Thr179Pro	novel	No data	−/−	−/−	−/−	−/−	−/−
*FSD1*	Chr19:4311959	c.608G>A	p.Arg203Gln	novel	No data	−/+	−/−	−/+	−/+	−/−
*LMNA*	Chr1:156134901	c.736C>T	p.Gln246Ter	rs267607587	No data	−/+	−/+	−/+	−/+	−/−
*OR51A7*	Chr11:4908132	c.763A>G	p.Ile255Val	rs144609747	0.0040	−/+	−/+	−/+	−/+	−/−
*SYPL2*	Chr1:109476793	c.272A>T	p.Tyr91Phe	rs79613472	0.0086	−/+	−/+	−/+	−/+	−/+

^a^ Variants shared by all four affected individuals (III-4, IV-2, IV-3, P3) and unshared with the one unaffected individual (IV-1) are highlighted in gray (*n* = 3). ^b^ Hyperlinked to GeneCards database (www.genecards.org). ^c^ Hyperlinked to dbSNP (www.ncbi.nlm.nih.gov/projects/SNP). ^d^ Hyperlinked to ExAC database (http://exac.broadinstitute.org). ^e^ Genotypes: −/+, heterozygous for variant allele; +/+ homozygous for variant allele; −/− homozygous for non-variant allele.

## Supplementary Materials

The following are available online at www.mdpi.com/2073-4409/6/4/40/s1, Table S1: Clinical features of study family, Table S2: Exome sequencing studies, Table S3: Candidate DNA variants.
