# Supplementary material for: Dupuytren’s and Ledderhose Diseases in a Family with LMNA-Related Cardiomyopathy and a Novel Variant in the ASTE1 Gene
Source: Cells. 2017 Nov 1;6(4):40. doi: 10.3390/cells6040040 (PMC5753070; doi:10.3390/cells6040040)
Supplement: Supplementary file 1 [file cells-06-00040-s001.pdf]

Table S1. Main clinical features of the study family.

ID[a]	Sex	Age (y)	Cardiac History (Age at Diagnosis)[b]	Dupuytren's Disease (Px Age)	Ledderhose Disease (Px Age)	Other Significant Medical and Social History
I-1	M	d.59	Unknown	Unknown	Unknown	Unknown
I-2	F	d.51	Sudden death (51)	Unknown	Unknown	Unknown
I-3	M	d.80	Unknown	Unknown	Unknown	Unknown
I-4	F	d.75	Unknown	Unknown	Unknown	Unknown
II-1	M	d.neonate	Unknown	Unknown	Unknown	Unknown
II-2	F	d.52	Sudden death (52)	Unknown	Unknown	Unknown
II-3	M	d.74	Unknown	Unknown	Unknown	Unknown
II-4	F	d.16	Unknown	Unknown	Unknown	Unknown
II-5	M	d.53	Sudden death (53)	Unknown	Unknown	Unknown
II-6	F	d.87	Unknown	Unknown	Unknown	Unknown
II-7	M	d.68	Irregular heart beat (40s), pacemaker (64), HF (66), cardiac arrest (68)	Unknown	Unknown	Unknown
II-8	F	d.79	None	Yes (40s), Surgery x1 (60)	Unknown	Emphysema, lung cancer (79), alcoholism, tobacco use
II-9	M	d.unknown	Unknown	Unknown	Unknown	Unknown
III-1	M	d.58	CAD, MI (31, 58)	No	Unknown	None
III-2	F	d.74	AF, DCM, HF, ICD (68), ASD-repair (73), VT (72), pulmonary HTN (73)	Yes (50s), Surgery x1 (60)	No	Myelodysplastic syndrome, chronic renal insufficiency
III-3	M	d.76	None	No	No	Brain tumor (76)
III-4	F	75	AFL (61), bradycardia, pacemaker (66), ICD (73), normal echo (73)	Yes (40s)	Yes (40s)	None
III-5	M	d.57	Irregular heart beat (50s), enlarged heart (55), sudden death, HF (57)	Yes (20s), Surgery x4	No	Alcohol binge drinking
III-6	F	80	Unknown	Unknown	Unknown	Unknown
IV-1	F	60	None, normal echo, holter studies (56)	No	No	None
IV-2	F	58	Syncope, SSS, pacemaker (45), AF, NSVT, AV block, DCM, ICD (55)	Yes (50)	Yes (55)	Hyperlipidemia
IV-3	F	55	Syncope x3 (50, 52, 53), bradycardia (50), normal echo (53)	No	Yes (51)	None, normal CK (50)
IV-4	F	49	Dyspnea, bradycardia, palpitations, mild DCM (42), PVCs, NSVT (47)	No	Plantar nodule?	None
IV-5	F	47	None, normal echo, EKG (37)	No	No	Hypothyroidism

[a]Individuals with cardiac disease (arrhythmia, ventricular dysfunction, sudden death) with or without Dupuytren's Disease are highlighted in yellow (n=10). The maternal grandmother with Dupuytren's Disease and no cardiac disease is highlighted in green.
[b]Clinical features for an additional family member was removed after consent to publish was declined.
Abbreviations: y, years old; Px, presentation; d., died; HF, heart failure; CAD, coronary artery disease; MI, myocardial infarction; AF, atrial fibrillation; DCM, dilated cardiomyopathy; ICD, implantable cardiac defibrillator; ASD, atrial septal defect; VT, ventricular tachycardia; HTN, hypertension; AFL, atrial flutter; SSS, sick sinus syndrome; NSVT, nonsustained ventricular tachycardia; PVC, premature ventricular contraction; CK, creatine kinase

Table S2. Exome Sequencing Studies of Four Family Members: Sequencing-Mapping & Variant Calling-Filtering

	Family Member				All Four Family Members: Average/Sample
	III-4	IV-3	Patient 3	IV-1	
**Hand/Feet Phenotype**	DD & LD	LD only	DD only	Unaffected	
**Sequencing-Mapping**					
Raw data (bp)	7,907,552,128	8,967,512,187	7,055,268,986	8,411,863,057	8,085,549,090
Reads-total	104,800,788	118,853,834	93,507,716	111,492,986	107,163,831
Reads mapped	104,136,200	118,129,101	92,924,198	110,810,461	106,499,990
% Reads mapped	99.4%	99.4%	99.4%	99.4%	99.4%
Mean coverage depth	78X	88X	72X	85X	81X
Mean coverage $\geq$ 30X	85.7%	87.5%	83.2%	86.7%	85.8%
Bases: % $\geq$ Q30	90.4%	90.5%	90.0%	90.6%	90.4%
Bases: Mean Quality Score	32.62	32.64	32.55	32.67	32.62
**Variant Calling-Filtering**					
Total unique variants	20,144	20,445	20,576	20,500	20,416
Heterozygous variants	12,555	12,894	13,093	12,955	12,874
HET/Non-synonymous variants	6,327	6,445	6,614	6,564	6,488
HET/NS/Highly-conserved variants	1,677	1,681	1,815	1,761	1,734

Abbreviations: DD, Dupuytren's Disease; LD, Ledderhose Disease; Q, Quality Score; HET, heterozygous; NS, non-synonymous

Table S3. Exome Sequencing Studies of Four Family Members: Candidate Variants by Allele Frequency (n=98)
Novel or rare (allele frequency <1%) variants considered as high-priority candidates are highlighted in yellow. The USF3 variant was excluded since it is an inframe-deletion of one codon and the clinical significance of the variant is likely benign (ClinVar variant # 218482).

Gene	Position (hg38)	DNA variant	Protein change	dbSNP (rs)	ExAC Frequency
ASTE1	Chr3:131025077	c.230T>C	p.Val77Ala	novel	no data
FZD2	Chr17:44558223	c.535A>C	p.Thr179Pro	novel	no data
FSD1	Chr19:4311959	c.608G>A	p.Arg203Gln	novel	no data
LMNA	Chr1:156134901	c.736C>T	p.Gln246Ter	267607587	no data
USF3	Chr3:113657264-113657266	c.4416_4418delGCA	p.Gln1478del	10606566	no data
OR51A7	Chr11:4908132	c.763A>G	p.Ile255Val	144609747	0.0040
SYPL2	Chr1:109476793	c.272A>T	p.Tyr91Phe	79613472	0.0086
CARD14	Chr17:80202245	c.2044C>T	p.Arg682Trp	117918077	0.0110
COL24A1	Chr1:86126053	c.283G>A	p.Val95Met	74097691	0.0127
OSGEPL1	Chr2:189755490	c.292G>A	p.Ala98Thr	75321854	0.0416
PEX11G	Chr19:7477282	c.436C>T	p.Leu146Phe	11668511	0.0488
OR10T2	Chr1:158398602	c.865G>A	p.Val289Ile	61818748	0.0504
MUC6	Chr11:1021268	c.3536A>G	p.Asn1179Ser	113451874	0.0524
DOCK6	Chr19:11214289	c.4324G>A	p.Ala1442Thr	34243815	0.0681
FAM221A	Chr7:23698272	c.544A>G	p.Ser182Gly	35928055	0.0791
COX4I1	Chr16:85801212	c.7G>A	p.Ala3Thr	11557187	0.0906
OR52L1	Chr11:5986542	c.389C>T	p.Ser130Leu	61750896	0.0965
RERGL	Chr12:18081322	c.484A>G	p.Met162Val	941048	0.0987
CAMTA1	Chr1:7737443	c.3531C>G	p.Asn1177Lys	41278952	0.1167
KANK3	Chr19:8325009-8325011	c.2022_2024delGGA	p.Glu674del	111905975	0.1298
OR52J3	Chr11:5047067	c.542G>A	p.Cys181Tyr	58664826	0.1303
NLRP14	Chr11:7038750	c.164G>A	p.Arg55Gln	61063081	0.1500
TLN2	Chr15:62819540	c.6796T>C	p.Phe2266Leu	3816988	0.1640
COL24A1	Chr1:86046853	c.1922G>A	p.Arg641His	60891279	0.1703
BAIAP2	Chr17:81115790	c.1556A>G	p.Gln519Arg	4969391	0.1823
RBMXL2	Chr11:7089317	c.197C>T	p.Ala66Val	11041170	0.2020
ACAN	Chr15:88872016	c.7005C>G	p.Asp2335Glu	3817428	0.2023
C5orf49	Chr5:7835442	c.204G>C	p.Gln68His	6883562	0.2134
SSC4D	Chr7:76400378	c.383G>A	p.Arg128His	4728712	0.2209

MATN4	Chr20:45297933	c.1564G>A	p.Gly522Ser	2227275	0.2300
PTCRA	Chr6:42923284	c.316G>A	p.Val106Ile	9471966	0.2383
PTPRG	Chr3:62203515	c.1720G>A	p.Gly574Ser	2292245	0.2385
OR13C9	Chr9:104617614	c.591C>A	p.Phe197Leu	10761054	0.2444
QRFPR	Chr4:121329579	c.1031T>C	p.Leu344Ser	2302310	0.2485
SPTA1	Chr1:158627717	c.5572C>G	p.Leu1858Val	3737515	0.2567
NRAP	Chr10:113633160	c.1556A>T	p.Asn519Ile	2270182	0.2638
GLMP	Chr1:156294149	c.667A>G	p.Ile223Val	10908495	0.2688
C21orf58	Chr21:46302071-46302072	c.578_579insCCA	p.His193_Ala194insHis	71318063	0.2852
HDGF	Chr1:156743766	c.650C>T	p.Pro217Leu	4399146	0.2854
KRT40	Chr17:40983118	c.458C>T	p.Thr153Met	9908304	0.2925
APOB	Chr2:21008652	c.8216C>T	p.Pro2739Leu	676210	0.2928
KRT40	Chr17:40983969	c.305G>A	p.Ser102Asn	1510068	0.2934
MED12L	Chr3:151372636	c.3629G>A	p.Arg1210Gln	3732765	0.3060
ST5	Chr11:8730093	c.1197C>G	p.Asp399Glu	3812762	0.3119
CALU	Chr7:128754552	c.245C>T	p.Ala82Val	2307040	0.3138
BRCA1	Chr17:43092418	c.3113A>G	p.Glu1038Gly	16941	0.3429
MYO19	Chr17:36498436	c.2587C>T	p.Leu863Phe	2306590	0.3462
SMG6	Chr17:2188471	c.190G>A	p.Ala64Thr	903160	0.3491
CSPG4	Chr15:75677729	c.5108G>A	p.Arg1703His	8023621	0.3496
ETV1	Chr7:13939184	c.298A>G	p.Ser100Gly	9639168	0.3500
NRAP	Chr10:113590838	c.4696C>T	p.Arg1566Cys	1885434	0.3535
RHPN2	Chr19:32999660	c.1151A>G	p.Gln384Arg	74582927, 201801079	0.3653
RHBG	Chr1:156377340	c.20G>A	p.Gly7Asp	2245623	0.3740
NAALADL2	Chr3:175096948	c.232G>A	p.Gly68Ser	9823911	0.3835
INO80D	Chr2:206046504	c.1073C>T	p.Ala358Val	2909111	0.3865
KRT72	Chr12:52592403	c.791A>G	p.Tyr264Cys	12833456	0.3870
DST	Chr6:56618003	c.6031G>C	p.Val2011Leu	6459166	0.3887
OR2G3	Chr1:247606450	c.865A>G	p.Ile289Val	61730407	0.3979
KRTAP4-8	Chr17:41098083-41098084	c.1_2insA	p.Met1Asnfs	201764113	0.4025
BRCA1	Chr17:43092919	c.2612C>T	p.Pro871Leu	799917	0.4100
WDR72	Chr15:53710894	c.917C>T	p.Pro306Leu	551225	0.4113
EFCAB6	Chr22:43687562	c.1051A>G	p.Thr351Ala	5764214	0.4235
MROH8	Chr20:37155091	c.1076A>G	p.Lys359Arg	1615246	0.4285
THSD7A	Chr7:11469934	c.2313C>G	p.Asp771Glu	2285744	0.4303

NBR1	Chr17:43209941	c.2768A>G	p.His923Arg	8482	0.4349
ATP13A5	Chr3:193276754	c.3392T>C	p.Val1131Ala	2271791	0.4362
MGAM	Chr7:142065775	c.4714A>T	p.Met1572Leu	4507684	0.4367
TMEM241	Chr18:23373756	c.28C>T	p.Leu10Phe	8099409	0.4377
TNC	Chr9:115084301	c.2039A>G	p.Gln680Arg	1061494	0.4408
HS6ST1	Chr2:128318303	c.261C>A	p.Asp87Glu	200979099	0.4625
LETMD1	Chr12:51049161	c.250G>A	p.Val84Ile	12379	0.4645
MROH8	Chr20:37112391	c.2777A>G	p.Gln926Arg	1744760	0.4781
PSPC1	Chr13:19703273	c.1474A>G	p.Met492Val	3852596	0.4797
RFX8	Chr2:101402446	c.1235T>C	p.Met412Thr	2175968	0.4965
WDR4	Chr21:42873634	c.213G>C	p.Lys71Asn	2248490	0.4968
ANKRD35	Chr1:145872788	c.1711C>G	p.Gln571Glu	11579366	0.5068
RB1CC1	Chr8:52674146	c.701T>C	p.Met234Thr	17337252	0.5110
MALRD1	Chr10:19331510	c.3829A>G	p.Ile1277Va	7100403	0.5211
HELQ	Chr4:83453327	c.916G>A	p.Val306Ile	1494961	0.5623
PCMTD1	Chr8:51820490	c.707A>T	p.Asn236Ile	12335014	0.5638
TNS2	Chr12:53049172	c.29G>C	p.Arg10Thr	12369033	0.5647
RGS9BP	Chr19:32676549	c.286G>T	p.Ala96Ser	259290	0.5674
TMEM185B	Chr2:120222852	c.125C>G	p.Ala42Gly	11550347	0.5829
LRP4	Chr11:46877220	c.3256A>G	p.Ile1086Val	6485702	0.5849
ABCA6	Chr17:69085689	c.3965A>G	p.Asn1322Ser	2302134	0.5867
RCBTB1	Chr13:49567209	c.71C>T	p.Ala24Val	4942848	0.6165
MRPL30	Chr2:99195607	c.388G>A	p.Ala130Thr	1044575	0.6366
PAPPA	Chr9:116344602	c.3671C>A	p.Ser1224Tyr	7020782	0.6687
C9orf129	Chr9:93335465	c.274G>A	p.Gly92Ser	3122944	0.7128
WDR91	Chr7:135204389	c.770C>T	p.Pro257Leu	292592	0.7135
GEN1	Chr2:17761508	c.274T>A	p.Ser92Thr	1812152	0.7138
EPB41L4A	Chr5:112184043	c.1595A>G	p.Asn532Ser	1560058	0.7349
ADGRE2	Chr19:14751618	c.1668G>C	p.Leu556Phe	2524383	0.7371
KRT74	Chr12:52571389	c.813A>C	p.Glu271Asp	670741	0.7449
OBSL1	Chr2:219556695	c.4095G>C	p.Glu1365Asp	1983210	0.7461
OR56B1	Chr11:5736832	c.316T>C	p.Cys106Arg	7397032	0.8171
ZZEF1	Chr17:4142807	c.89T>C	p.Val30Ala	1454121	0.8454
BRDT	Chr1:91962938	c.184C>A	p.Gln62Lys	10783071	0.8763
